# Supplementary material for: The determinants of handwashing behaviour among internally displaced women in two camps in the Kurdistan Region of Iraq
Source: PLoS One. 2020 May 8;15(5):e0231694. doi: 10.1371/journal.pone.0231694 (PMC7209201; doi:10.1371/journal.pone.0231694)
Supplement: S1 File — (DOCX) [file pone.0231694.s001.docx]

Group: ❑ Doer ❑ Non-Doer

**Barrier Analysis Questionnaire:**

**Hand Washing among Mothers**

**Behavior Statement**

Mothers with children under the age of 5yrs who wash their hands with soap

at the five critical times each day.

**Demographic Data**

Questionnaire No.: ______ Date____/____/____

Community/camp: ____________________________

**Section A. Behavior Screening Questions**

1. How old is your youngest child? _________years _________ months

**€** a. 0 months - <5 years

**€** b. >5 years 🡪 *End interview and look for another respondent*

1. Yesterday, did you wash your hands?

**€** a. Yes

**€** b. No *🡪 Mark as Non-doer and continue to Section B*

**€** c. Don’t remember 🡪 *End interview and look for another respondent*

1. Yesterday, what are all the moments that you washed your hands? (DO NOT READ THE LIST – Mark all that are mentioned)

€ a. after defecation

€ b. after cleaning a child’s diaper/nappy

€ c. before cooking / preparing food

€ d. before eating

€ e. before feeding a child

€ f. Don’t know or won’t say🡪 *End interview and look for another respondent*

1. In addition to water, did you use anything else to wash your hands yesterday?

**€** a. Yes

**€** b. No 🡪 *Mark as Non-doer and continue to Section B*

**€** c. Don’t remember 🡪 *End interview and look for another respondent*

1. In addition to water, what else did you use to wash your hands?

€ a. Soap

€ b. Anything else 🡪 *Mark as Non-doer and continue to Section B*

€ c. Don’t know/refused to answer 🡪 *End interview and look for another respondent*

1. May I see the soap that you use?

€ a. Soap available and looks used

€ b. Soap available but does not look used 🡪 *Mark as Non-doer and continue to Section B*

€ c. No soap available🡪 *Mark as Non-doer and continue to Section B*

1. Where do you normally keep this soap?

€ a. Soap kept at the handwashing facility or near the toilet and/or kitchen

€ b. Soap kept elsewhere 🡪 *Mark as Non-doer and continue to Section B*

| **Doer**  (all of the following) | **Non Doer**  (any one of the following) | **Do not Interview**  (any one of the following) |
| --- | --- | --- |
| Question 1 - A |  | Question 1 -C |
| Question 2 - A | Question 2 – B | Question 2 – C |
| Question 3 - **A plus** any **two** from B, C, D, E | Question 3 – No A; or  A and **only one** other response between B, C, D, E | Question 3 -C |
| Question 4 – A | Question 4 – B | Question 4 - C |
| Question 5 – A | Question 5 - B | Question 5 - C |
| Question 6- A | Question 6- B or C |  |
| Question 7- A | Question 7 - B |  |

**GROUP: € DOER € NON-DOER**

**Behavior Explanation:** In the following questions I am going to be talking about hand washing with soap *at five critical times*. By this I mean 1. after defecation, 2. after changing a baby’s diaper/nappy, 3. before cooking, 4. before eating and 5. before feeding a child.

**Section B – Research Questions**

*(Perceived Self-efficacy)*

**1**. With your current knowledge, skills and resources do you think you can wash your hands with soap at the five critical times?

❑ a. Yes

❑ b. No

❑ c. Maybe

❑ d. Don’t know/ Won’t say

**2a. *Doers****: What makes it* ***easier*** *for you to wash your hands with soap at the five critical times each day.*

**2b.** ***Non-doers****: What would make it* ***easier*** *for you to wash your hands with soap at the five critical times each day.*

*(Write all responses below. Probe with “What else?”)*

*(Perceived Self-efficacy)*

**3a. *Doers****: What makes it* ***difficult*** *for you to washing your hands with soap at the five critical times each day.*

**3b.** ***Non-doers****: What would make it* ***difficult*** *for you to washing your hands with soap at the five critical times each day.*

*(Write all responses below. Probe with “What else?”)*

*(Perceived Positive Consequences)*

**4a.** ***Doers:*** What are the ***advantages*** of washing your hands with soap at the five critical times each day?

**4b. *Non-doers:*** What would be the ***advantages*** of washing your hands with soap at the five critical times each day?

*(Write all responses below. Probe with “What else?”)*

*(Perceived Negative Consequences)*

**5a.** ***Doers:*** What are the ***disadvantages*** of washing your hands with soap at the five critical times each day?

**5b. *Non-doers:*** What would be the ***disadvantages*** of washing your hands with soap at the five critical times each day?

*(Write all responses below. Probe with “What else?”)*

*(Perceived Social Norms )*

**6a.** ***Doers:*** Who are the people that ***approve*** of you washing your hands with soap at the five critical times each day.

**6b.** ***Non-doers:*** Who are the people that ***would approve*** of you washing your hands with soap at the five critical times each day.

*(Write all responses below. Probe with “Who else?”)*

*(Perceived Social Norms )*

**7a. *Doers:*** Do most of the people that you know approve of you washing your hands with soap at the five critical times each day?

**7b.** ***Non-doers***: Would most of the people that you know approve of you washing your hands with soap at the five critical times each day?

❑ a. Yes

❑ b. Possibly

❑ c. No

❑ d. Don’t Know / Won’t say

*(Perceived Social Norms )*

**8a.** ***Doers:*** Who are the people that ***disapprove*** of you washing your hands with soap at the five critical times each day.

**8b.** ***Non-doers:*** Who are the people that ***would disapprove*** of washing your hands with soap at the five critical times each day.

*(Write all responses below. Probe with “Who else?”)*

*(Perceived Access)*

**9a.** ***Doers:*** How difficult is it to get the soap you need to wash your hands at the five critical times each day? Would you say it is very difficult, somewhat difficult or not difficult at all?

**9b. *Non-doers:*** How difficult would it be to the water soap needed to wash your hands at the five critical times each day? Would you say it is: Very difficult, somewhat difficult, not difficult at all?

❑ a. Very difficult

❑ b. Somewhat difficult

❑ c. Not difficult at all.

*(Perceived Cues for Action / Reminders)*

**10a. *Doers:*** How difficult is it to remember to wash your hands with soap at the five critical times each day? Very difficult, somewhat difficult, or not difficult at all?

**10b. *Non-doers:*** How difficult do you think it would be to remember to wash your hands with soap at the five critical times each day? Very difficult, somewhat difficult, or not difficult at all?

❑ a. Very difficult

❑ b. Somewhat difficult

❑ c. Not difficult at all.

❑ d. Don’t Know / Won’t say

*(Perceived Susceptibility / Perceived Risk)*

**11.** ***Doers* and Non-doers:** How likely is it that your child will get diarrhea in the coming 3 months ? Very likely, somewhat likely, or not likely at all?

❑ a. Very likely

❑ b. Somewhat likely

❑ c. Not likely at all

*(Perceived Severity)*

**12.** **Doers and Non-doers:** How serious would it be if your child got diarrhea? A very serious problem, somewhat serious problem, or not serious at all?

❑ a. Very serious problem

❑ b. Somewhat serious problem

❑ c. Not serious at all

*(Action Efficacy)*

**13. Doers and Non-doers** How likely is it that your child will suffer from diarrhea if you wash your hands with soap at the five critical times each day? Very likely, somewhat likely, not very likely?

❑ a. Very likely

❑ b. Somewhat likely

❑ c. Not likely at all

*(Perception of Divine Will)*

**14a.** ***Doers and Non-doers:***  Do you think that it’s **God will** that children get diarrhea?

❑ a. Yes

❑ b. Sometimes/situation dependent

❑ b. No

❑ c. Don’t Know / Won’t say

*(Culture)*

**15. Doers and Non-doers:** Are there any cultural rules or taboos against washing your hands with soap at the five critical times each day. ?

❑ a. Yes

❑ b. No

❑ c. Don’t Know / Won’t say

*(Policy)*

**16**. **Doers and Non-doers** : Are there any community laws or rules in place that make it more likely that you wash your hands with soap at the five critical times each day.

❑ a. Yes

❑ b. No

❑ c. Don’t Know / Won’t say

***THANK THE RESPONDENT FOR HIS OR HER TIME!***
